# Multifaceted Intervention by the Hsp90 Inhibitor Ganetespib (STA-9090) in Cancer Cells with Activated JAK/STAT Signaling

**DOI:** 10.1371/journal.pone.0018552

**Published:** 2011-04-14

**Authors:** David A. Proia, Kevin P. Foley, Tim Korbut, Jim Sang, Don Smith, Richard C. Bates, Yuan Liu, Alex F. Rosenberg, Dan Zhou, Keizo Koya, James Barsoum, Ronald K. Blackman

**Affiliations:** Synta Pharmaceuticals Corp., Lexington, Massachusetts, United States of America; Ludwig-Maximilians University, Germany

## Abstract

There is accumulating evidence that dysregulated JAK signaling occurs in a wide variety of cancer types. In particular, mutations in JAK2 can result in the constitutive activation of STAT transcription factors and lead to oncogenic growth. JAK kinases are established Hsp90 client proteins and here we show that the novel small molecule Hsp90 inhibitor ganetespib (formerly STA-9090) exhibits potent *in vitro* and *in vivo* activity in a range of solid and hematological tumor cells that are dependent on JAK2 activity for growth and survival. Of note, ganetespib treatment results in sustained depletion of JAK2, including the constitutively active JAK2^V617F^ mutant, with subsequent loss of STAT activity and reduced STAT-target gene expression. In contrast, treatment with the pan-JAK inhibitor P6 results in only transient effects on these processes. Further differentiating these modes of intervention, RNA and protein expression studies show that ganetespib additionally modulates cell cycle regulatory proteins, while P6 does not. The concomitant impact of ganetespib on both cell growth and cell division signaling translates to potent antitumor efficacy in mouse models of xenografts and disseminated JAK/STAT-driven leukemia. Overall, our findings support Hsp90 inhibition as a novel therapeutic approach for combating diseases dependent on JAK/STAT signaling, with the multimodal action of ganetespib demonstrating advantages over JAK-specific inhibitors.

## Introduction

JAK2 is a ubiquitously expressed member of the Janus-associated kinase (JAK) family of non-receptor tyrosine kinases which function to mediate signaling downstream of cytokine and growth factor receptors [1]. Inappropriate activation of JAK signaling underlies cell proliferation and survival in a variety of solid tumors [2], [3] and hematological neoplasms [4]. In particular, an activating point mutation in JAK2 (JAK2^V617F^) has been described with high frequency in chronic myeloproliferative disorders (MPD) [5], [6], [7], [8], [9], [10] and constitutive JAK2 activation caused by chromosomal translocations has been reported in various types of leukemia [11], [12], [13].

Activated cytokine-JAK complexes recruit and phosphorylate effector molecules including Signal Transducers and Activators of Transcription (STAT) proteins [14]. STAT proteins mediate a wide range of biological processes, including cell growth, differentiation, apoptosis, inflammation and immune response [15]. Two STATs in particular, STAT3 and STAT5, represent the major substrates for JAK2 that govern myelopoeisis [16], [17] and can contribute to cellular transformation [18], [19]. Their persistent activation has been linked to increased tumor cell proliferation, survival, metastasis and tumor-promoting inflammation in both solid and hematological tumors [20]. Accordingly, inhibiting this signaling axis by the use of specific small molecule inhibitors of JAK2 has recently been investigated as a point of therapeutic intervention in multiple human tumor indications [3], [21], [22], [23], [24].

Heat shock protein 90 (Hsp90) is a molecular chaperone required for the post-translational stability of its protein substrates or “client proteins”. Cancer cells contain elevated levels of active Hsp90 [25] and, because many client proteins play critical oncogenic roles, cancer cells are particularly sensitive to Hsp90 inhibition. Moreover, a unique characteristic of targeting Hsp90 is that inhibition results in the simultaneous blockade of multiple oncogenic signaling cascades, overcoming potential pathway redundancies, and sensitizing cancer cells to chemotherapeutic agents [26], [27], [28], [29]. Thus, Hsp90 represents an attractive molecular target for the development of novel cancer therapeutics [28], [30], [31]. Of relevance here, JAK kinases are established Hsp90 clients suggesting that Hsp90 inhibition may be effective in treating JAK-directed neoplastic disorders [32], [33].

To test this hypothesis, we have employed the synthetic small molecule inhibitor ganetespib (STA-9090), a resorcinol-containing compound unrelated to geldanamycin, that binds in the ATP-binding domain of Hsp90. In preclinical studies, the drug has shown low nanomolar activity *in vitro* against a variety of human cancer cell lines and potent antitumor efficacy against human xenograft models [34], [35]. Ganetespib is currently in clinical trials for both solid tumor and hematological malignancies. Here we show that ganetespib potently induces apoptosis in a variety of tumor lines dependent on persistent JAK/STAT signaling for growth and survival. We further demonstrate that the drug also alters many elements of cell cycle regulation in cancer cells, an activity absent from a JAK-specific inhibitor. *In vivo*, ganetespib's coordinate impact on both cell growth and cell division results in potent antitumor activity in JAK/STAT-driven models of human leukemia. Thus, inhibition of Hsp90 activity represents a promising approach for combating diseases dependent on constitutive JAK/STAT signaling, with ganetespib demonstrating potential therapeutic advantages over JAK-specific inhibitors.

## Results

### Ganetespib inhibits JAK2-mediated signal transduction and proliferation in hematological cancers

**Figure 1:**
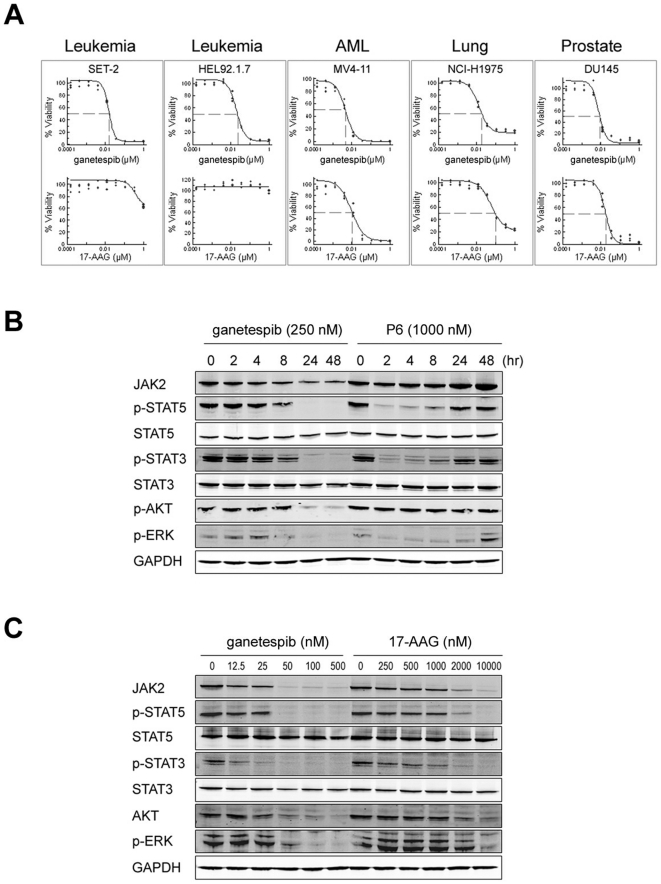
Effects of ganetespib on tumor cell viability. (A) SET-2, HEL92.1.7, MV4-11, NCI-H1975 and DU145 cells were treated with ganetespib or 17-AAG over a broad dose range (0.0001 to 1 uM) for 72 h and cell viability assessed by Alamar blue staining. (B) Ganetespib exhibits more durable inhibition of JAK/STAT signaling compared to P6. HEL92.1.7 cells were cultured in the presence of 250 nM ganetespib or 1000 nM P6 and harvested between 0 and 48 h. Expression levels of the indicated proteins were determined by western blot. (C) Ganetespib is significantly more potent than 17-AAG. SET-2 cells were dosed with the indicated concentrations of ganetespib or 17-AAG for 24 h and analyzed to determine JAK/STAT protein and target levels using the antibodies indicated.

With low nanomolar potency, ganetespib reduced cellular viability in a group of human hematological and solid tumor cell lines selected for their dependence on JAK/STAT signaling and varying cancer type (Fig. 1A). In each of the lines tested, ganetespib was more potent than the ansamycin Hsp90 inhibitor 17-AAG. Of note, ganetespib was greater than 100 fold more potent than 17-AAG in the SET-2 and HEL92.1.7 leukemia cells, cell lines harboring constitutively active JAK2^V617F^ mutations that act as their oncogenic drivers.

Using the HEL92.1.7 cells, we compared the JAK/STAT inhibitory activity of ganetespib to the compound Pyridone-6 (P6) [36], a reversible, ATP-competitive pan inhibitor of the JAKs (Fig. 1B). Ganetespib and P6 each blocked JAK2 dependent signaling, as evidenced by the loss of phospho-STAT3 and phospho-STAT5, and ERK signaling. However, ganetespib was at least four fold more potent and suppressed STAT signaling longer compared to P6. Ganetespib treatment alone led to the targeted, proteasome-dependent (data not shown) loss of JAK2 and phospho-AKT protein levels (Fig. 1B), both Hsp90 client proteins. Thus, ganetespib treatment results in sustained inhibition of multiple oncogenic targets in these cellular models of JAK2-driven malignancy. Similar effects on JAK/STAT signaling were seen with SET-2 cells, where 50 nM ganetespib was able to destabilize JAK2 sufficiently to result in loss of activated (*i.e.*, phosphorylated) STAT3 and STAT5 expression (Fig. 1C). 17-AAG showed comparable effects as ganetespib but was 200 fold less potent, in line with the viability data described above. Taken together, these data demonstrate that ganetespib possesses superior JAK/STAT inhibitory activity to both P6 and 17-AAG in terms of potency or duration of response.

### Ganetespib abrogates JAK/STAT signaling in solid tumors

**Figure 2:**
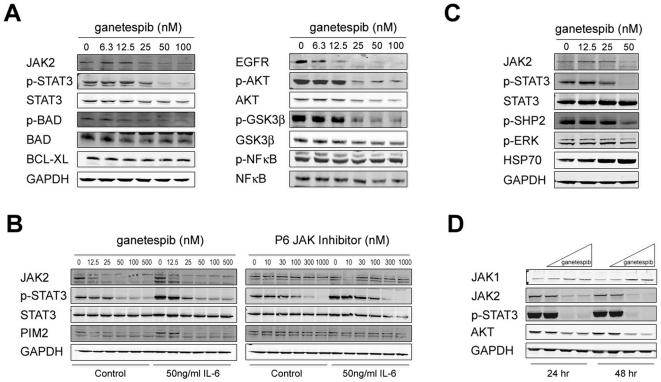
Inhibition of JAK2/STAT signaling by ganetespib in solid tumors. (A) Client protein downregulation in NSCLC. NCI-H1975 cells were dosed with the indicated concentrations of ganetespib for 24 h and their cell lysates analyzed to determine JAK/STAT and Hsp90 client protein levels using the antibodies indicated. (B) Ganetespib blocks IL-6 induced and constitutive STAT3 activity in NSCLC cells. HCC827 lung cancer cells were treated with increasing concentrations of ganetespib or P6 for 24 h followed by a 15 min stimulation with or without 50 ng/ml human recombinant IL-6. The levels of JAK2, total and phospho-STAT3, and PIM2 were analyzed by western blot. GAPDH is included as a loading control. (C) Client protein degradation in prostate cancer cells. DU145 cells were dosed with graded concentrations of ganetespib for 24 h and cell lysates subject to western blot to determine JAK/STAT and target protein levels using the antibodies indicated. (D) Functional Hsp90 is required for JAK2, but not JAK1, stability in DU145 cells. DU145 cells were treated with DMSO (control, C), 15 nM, 60 nM or 240 nM ganetespib for either 24 or 48 h and lysates probed by western blot with the indicated antibodies.

In addition to its incidence in hematologic malignancies, oncogenic STAT activation is also prevalent in a range of solid tumors. For example, persistently activated STAT3 is found in 50% of lung adenocarcinomas and is primarily observed in tumors harboring mutations in the epidermal growth factor receptor (EGFR) [37], [38]. The NCI-H1975 non-small cell lung cancer (NSCLC) cell line expresses the Hsp90 client EGFR^L858R/T790M^, a constitutively activated and erlotinib-resistant form of EGFR, and ganetespib treatment resulted in a dose-dependent decrease in EGFR expression in these cells (Fig. 2A). Moreover, ganetespib also induced potent degradation of JAK2 and loss of phosphorylated STAT3 in a dose-dependent manner. Inactivation of AKT and GSK3β, proteins important in regulating apoptosis, was observed with a similar dose response to that of JAK2/STAT3 signaling. Recently it was shown that JAK2 can modulate the activity of additional apoptotic regulators such as BAD and BCL-XL to promote cell survival [21]. Consistent with this, we detected a concomitant reduction in the levels of phosphorylated BAD (Fig. 2A), thus reducing the pro-apoptotic activity of this protein. These data suggest a potential mechanism to account for the cytotoxic response observed with ganetespib treatment (Fig. 1A).

The JAK/STAT signaling axis is a key modulator of cytokine signaling and one proposed mechanism for aberrant STAT3 activation in lung cancer involves the upregulation of autocrine and/or paracrine IL-6 signaling [2]. Therefore, we investigated whether ganetespib could inhibit this signaling in NSCLC cells. In the absence of external ligand, HCC827 cells treated with ganetespib exhibited a dose-dependent decrease in JAK2 expression, leading to a loss of STAT3 activity and expression of the downstream STAT target PIM2 (Fig. 2B). Biochemical inhibition of JAK2 by P6, albeit at higher concentrations, similarly down-regulated constitutive STAT3 activity but did not influence total JAK2 protein levels. Similarly, both compounds blocked JAK/STAT signaling stimulation when the pathway was activated by exogenous IL-6 treatments (Fig. 2B).

Dysregulated IL-6/JAK2 signaling has also been implicated in prostate cancer tumorigenesis [39], [40]. The DU145 prostate cancer cell line expresses an autocrine IL-6 signaling loop [41] and was recently reported to be sensitive to the effects of a novel small molecule JAK2 inhibitor *in vitro* and *in vivo* [3], [41]. Ganetespib was a potent inducer of cell death in this line (Fig. 1A). Biochemical characterization of DU145 cells revealed similar inhibitory effects on JAK2 signaling following ganetespib treatment (Fig. 2C). Loss of JAK2, phospho-STAT3 and phospho-SHP2, a JAK2 interacting phosphatase important for JAK2 signal transduction, was observed following addition of ganetespib. Interestingly, the related JAK1 kinase expressed in this cell line was not targeted for degradation but instead appeared to increase following ganetespib exposure (Fig. 2D). Similar results were obtained for the PC-3 prostate cancer cell line (data not shown). These data show that selective degradation of JAK2 in DU145 prostate cells was sufficient to abrogate subsequent activation of STAT3 signaling.

### Hsp90 inhibition downregulates transcription of JAK/STAT signaling targets and cell cycle genes

**Figure 3:**
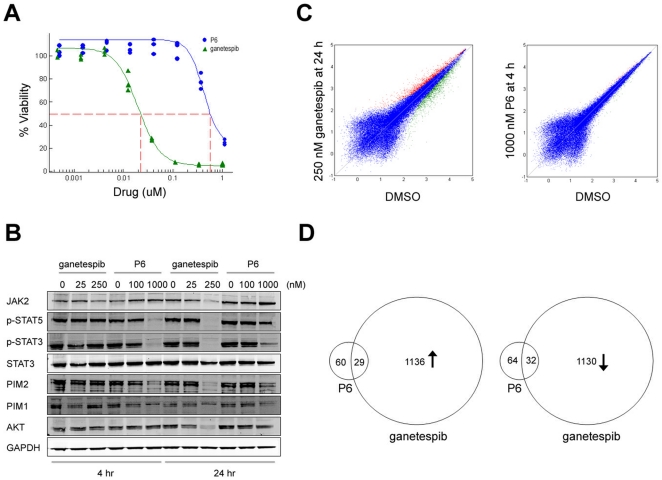
Ganetespib inhibits JAK/STAT target and cell cycle gene expression. (A) Comparative effects of ganetespib and P6 on HEL92.1.7 tumor cell viability. HEL92.1.7 cells were treated with ganetespib or P6 over a broad dose range (0.0001 to 10 uM) for 72 h and cell viability assessed by Alamar blue. (B) Temporal and dose-dependent effects on JAK/STAT targets by ganetespib and P6. HEL92.1.7 cells were treated with ganetespib or P6 for 4 and 24 h and cell lysates subject to western blot to determine JAK2/STAT and target protein levels using the indicated antibodies. (C) Affymetrix GeneChip analysis of cells treated with ganetespib and P6. HEL92.1.7 cells treated with 250 nM ganetespib for 24 h or 1000 nM P6 for 4 h. Gene expression levels in DMSO treated (i.e. vehicle control) cells (X-axis) are graphed against those of drug treated cells (Y-axis). (D) Venn diagram of number of genes differentially regulated by ganetespib and P6.

In HEL92.1.7 erythroleukemia cells, biochemical inhibition of JAK2 by P6 treatment resulted in a loss of cellular viability, but with 30 fold less potency than ganetespib (IC_50_ values 600 vs. 20 nM) (Fig. 3A). To compare the cellular impact of each inhibitor, we first identified conditions under which JAK2 activity was reduced to equivalent levels by each drug based on their kinetic and potency differences. As illustrated in Figure 3B, the 4 hour P6 (1000 nM) and 24 hour ganetespib (250 nM) treatments were selected because of comparable effects on STAT3/5 signaling. RNA expression profiling at these time points revealed, as expected, that many JAK/STAT target genes (such as SOCS and PIM family members) were downregulated by both drugs (Fig. S1, Table S1). However, additional genes were altered by ganetespib treatment that were unaffected in the P6-treated cells (Fig. 3 C,D). Besides leading to the expected upregulation of numerous heat shock protein genes, ganetespib treatment also selectively altered the expression of a large set of genes involved in cell cycle-related activities, including DNA replication and repair (BRCA1/2), cell cycle regulation (CDC2, CDC25), centrosome/spindle activities (BUB1/3, CENPE/M, KIF14, FAM33A), chromosome condensation (TOP2A, NCAPG), and replication (RFC3/4, MCM family) (Fig. S1). Indeed, analysis of the altered genes by hierarchical clustering and enrichment score revealed that modulators of cell division were the most prominent processes diminished by ganetespib treatment (Table 1).

**Table 1:** Gene groupings negatively regulated by ganetespib treatment tabulated according to hierarchical clustering and enrichment score analysis.

Annotation Cluster	Terms	Enrichment Score
cell cycle/division, mitosis	cell cycle, cell cycle process, M phase, mitosis, cell/nuclear division, organelle fission	61
DNA replication	DNA replication, DNA metabolic process	45
DNA repair/response to DNA damage, stress response	DNA repair, response to DNA damage stimulus, cellular response to stress	29
microtubule cytoskeletal and spindle organization	spindle organization, microtubule based process, microtubule cytoskeletal organization, cytoskeletal organization	17
RNA processing/binding	RNA processing/binding	12
ribosome biogenesis, rRNA and ncRNA processing	ribosome biogenesis, ncRNA processing/metabolic processing, rRNA/metabolic processing, ribonucleoprotein complex biogenesis	11
cell cycle checkpoint and regulation	cell cycle checkpoint, regulation of: cell cycle, mitosis, nuclear division, organelle organization	11
meiosis	meiotic cell cycle, meiosis, M phase of cell cycle	10
DNA damage response and checkpoint	cell cycle checkpoint, DNA integrity checkpoint, DNA damage checkpoint, DNA damage response	9
chromosome segregation and localization	chromosome segregation, mitotic sister chromatic segregation, chromosome localization, metaphase plate congression, organelle localization	9
nuclear envelope/pore, RNA transport	organelle envelope, nuclear envelope, envelope translocation, nuclear pore/complex, RNA localization, nucleic acid/RNA/mRNA transport, endomembrane system	8
DNA replication and repair	DNA replication, replication fork, mismatch repair, nucleotide excision repair/gap filling, protein-DNA loading ATPase activity, DNA clamp loader activity, replication factor C	8
RNA splicing and mRNA processing	RNA splicing, nuclear mRNA splicing, mRNA processing/metabolic processing, spliceosome	6

### Modulation of cell cycle protein expression by ganetespib induces growth arrest

**Figure 4:**
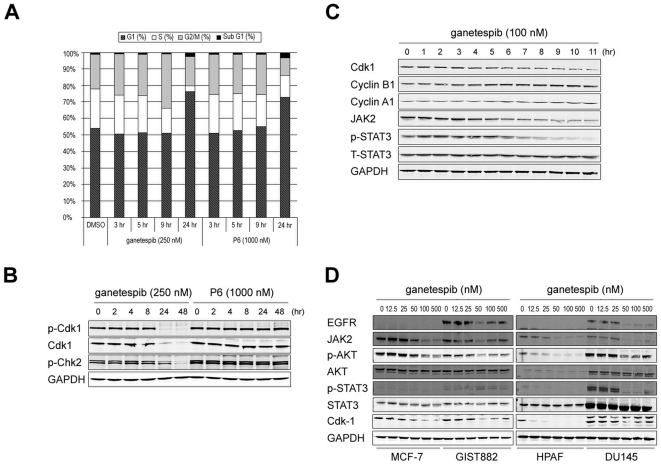
Ganetespib modulates cell cycle protein expression and induces growth arrest. (A) HEL92.1.7 cells were treated with 250 nM ganetespib or 1000 nM P6 (or DMSO as a control) and cell cycle distribution determined by flow cytometry at 3, 5, 9 and 24 h post-treatment. (B) HEL92.1.7 cells were dosed with ganetespib (250 nM) or P6 (1000 nM) for 48 h. Cells were harvested at the indicated time points and the levels of total and phospho-Cdk1, phospho-Chk2 and GAPDH analyzed by western blot. (C) Kinetics of ganetespib effects on JAK/STAT and cell cycle protein expression. HEL92.1.7 cells were treated with 100 nM ganetespib, harvested at hourly intervals over an 11 h time course, and subject to western blot with the indicated antibodies. (D) MCF-7, GIST882, HPAF and DU145 cells were dosed with graded concentrations of ganetespib for 24 h and analyzed by western blot using the indicated antibodies.

To extend these findings, we looked experimentally at the effects on the cell cycle. Ganetespib induced a temporal G_1_ and G_2_/M arrest in HEL92.1.7 cells, with concomitant loss of S phase (Fig. 4A). In contrast, P6 treatment induced accumulation in G_1_ phase only, without the loss of S phase or G_2_/M arrest. We also examined the targeted effects of ganetespib on critical mediators of cell cycle division at the protein level. We observed reduced protein levels of cyclin dependent kinase 1 (Cdk-1), a key regulator of the G_2_/M checkpoint, following a 24 hour exposure to ganetespib, an effect that persisted until at least 48 hours (Fig. 4B). In contrast, P6 had no effect on Cdk1 expression. Further, the level of phospho-Chk2, another integral checkpoint kinase, was reduced by ganetespib treatment. Similar results were found for phospho-Chk1 (data not shown). As shown in Figure 4C, the destabilization of cyclin kinases was also associated with a temporal accumulation of cyclins A1 and B1 in response to drug addition. Moreover, these effects of ganetespib on both JAK2/STAT signaling and cell cycle regulation were observed in additional cancer types, including breast (MCF-7), gastrointestinal stromal (GIST882), pancreatic (HPAF) and prostate (DU145) tumor cell lines (Fig. 4D). Overall, these additional influences on the cell division machinery suggest that ganetespib possesses decided advantages over JAK-specific inhibitors for controlling STAT-driven malignancies.

### Ganetespib prolongs survival in a JAK2^V617F^-mutant mouse model of human leukemia

**Figure 5:**
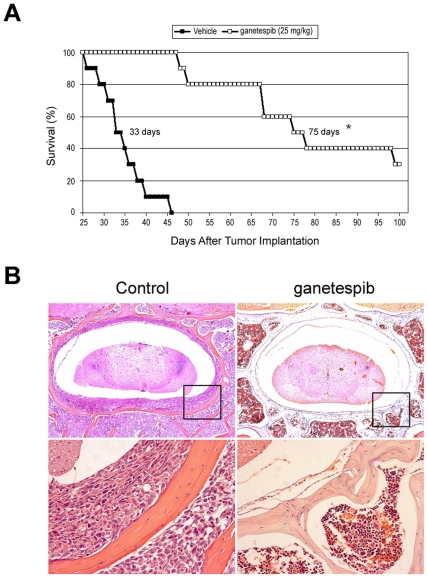
Ganetespib is highly efficacious *in vivo* in a leukemia survival model expressing activated JAK2^V617F^. (A) Kaplan-Meier analysis of overall survival in a leukemia model established by i.v. injection of HEL92.1.7 cells into SCID mice, which resulted in the development of disseminated disease. Beginning one day after tumor cell implantation, ganetespib was i.v. dosed at its HNSTD (25 mg/kg) on a five-times per week schedule for 3 weeks through day 19 (n = 10/group). *P<0.0001; 2-sided log-rank test. (B) Ganetespib dramatically inhibits tumor cell burden in the spinal cord and adjacent bone marrow. Immunohistochemistical staining (H&E) of lumbar spine cross sections from vehicle control (left panels) or ganetespib treated (right panels) animals. Insets are enlarged in the lower panels. Original magnification: 40× in upper panels; 200× in the lower panels.

To determine whether these dual activities of ganetespib on JAK2/STAT signaling and cell cycle progression observed *in vitro* translate into antitumor efficacy *in vivo*, we established an orthotopic leukemia model using HEL92.1.7 cells. This resulted in disseminated disease with morbidity typically resulting from hind limb paralysis caused by spinal column metastases (data not shown). To study the effect of ganetespib on survival, beginning one day after tumor cell implantation, the drug was dosed intravenously at its highest non-severely toxic dose (HNSTD) of 25 mg/kg on a 5×/week schedule through day 19. As shown in Figure 5A, ganetespib treatment more than doubled median overall survival (76.5 days *vs.* 34 days, *P*<0.0001). The ganetespib treatment was well tolerated, with no significant loss of body weight found after 3 weeks of dosing (Fig. S2). The increased survival of the treated animals correlated with dramatically decreased tumor cell burden in their bone marrow and spinal cord, as determined by histological analysis (Fig. 5B).

### Ganetespib exhibits potent *in vivo* efficacy in STAT5 driven AML xenografts

**Figure 6:**
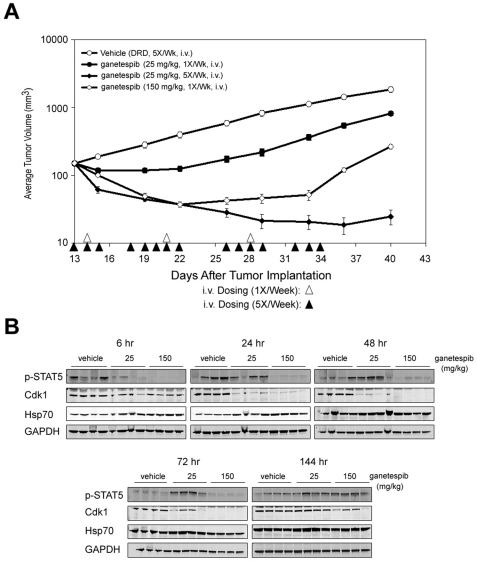
Ganetespib efficacy and pharmacodynamics in an *in vivo* leukemia model with constitutively activated STAT5 signaling. (A) SCID mice were subcutaneously implanted with MV4-11 acute myeloid leukemia cells. Mice bearing established MV4-11 xenografts (100–200 mm^3^, n = 8 mice/group) were i.v. dosed (arrowheads) with ganetespib at either 25 or 150 mg/kg once weekly for 3 weeks, or at the HNSTD of 25 mg/kg five-times per week, as indicated. % T/C values are indicated to the right of each growth curve and the error bars are the s.e.m. (B) ganetespib inhibits STAT-5 phosphorylation and Cdk1 expression in tumor xenografts in SCID mice. SCID mice bearing MV4-11 tumors (n = 4 mice/group) were treated with vehicle or ganetespib at either 25 mg/kg or 150 mg/kg at the indicated time points between 6 h and 144 h (6 days). Tumors were resected and the levels of p-STAT5, Cdk1, Hsp70 and GAPDH were determined by western blot.

MV4-11 acute myeloid leukemia cells express constitutive STAT5 activity as a consequence of an internal tandem duplication (ITD) mutation in the FLT3 receptor tyrosine kinase, another Hsp90 client protein [42] and, as such, represent an alternative model of STAT-driven oncogenesis. These cells are highly sensitive to ganetespib *in vitro* (Fig. 1A) and we evaluated their dose-response to ganetespib treatments in xenografts. Ganetespib was intravenously administered to tumor-bearing SCID mice at either the daily or weekly HNSTD of 25 mg/kg or 150 mg/kg, respectively. As shown in Figure 6A, the weekly treatment schedule resulted in significant and dose-dependent tumor growth inhibition, while the daily dosing regimen (25 mg/kg 5×/week, as used in the orthotopic model above) resulted in significant tumor regression (84%). In both dosing regimens, tumor growth was suppressed for up to a week or more once treatment was discontinued. Beyond this period, as evidenced by the once-per-week treatment cohort, tumor growth could re-initiate.

To determine whether these tumor responses correlated with target modulation *in vivo*, additional mice bearing MV4-11 xenografts were treated with a single dose of vehicle alone or ganetespib at 25 or 150 mg/kg. Tumors were harvested between 6 and 144 hours later and pharmacodynamic analysis was performed by examining the expression levels of phospho-STAT5, Cdk1 and Hsp70 (Fig. 6B). In accord with the *in vivo* tumor growth data, dose-dependent effects on the duration of target inhibition within the tumors were observed. A single 150 mg/kg dose of ganetespib repressed activation of STAT5 and suppressed expression of Cdk1 for more than three days, consistent with its efficacy in once-per-week dosing. At 25 mg/kg, potent inhibition of STAT5 activity was achieved within 6 hours as was loss of Cdk1 at 24 hours following ganetespib administration. Of note, STAT5 activity recovered in these tumors by 24 hours, while Cdk1 expression remained suppressed through at least 48 hours even with this low dose (Fig. 6B). While the relatively quick recovery of STAT5 activation should have allowed the tumor to restart growth, the more durable suppression of the cell cycle regulators appears to have kept the growth of the tumors arrested until the next drug dosing. These findings provide strong evidence that the coordinate loss of cell growth and cell division signals orchestrated by ganetespib account for the potent antitumor activity of the drug in this model.

## Discussion

Persistent JAK/STAT activation is oncogenic and characteristic of many human malignancies and thereby provides an attractive point of intervention for molecularly targeted therapeutics. In this study, we show that ganetespib has profound antitumor activity in an array of JAK/STAT-driven cancers and, importantly, can abrogate aberrant signaling through multiple mechanisms. Ganetespib effectively targets the upstream regulator JAK2, including the constitutively active JAK2^V617F^ mutant, for degradation in a range of hematological and solid tumor types with subsequent prolonged loss of STAT3 and STAT5 signaling. The findings not only underscore the pathogenic role of STAT signaling in tumorigenesis, but support the potential therapeutic utility of ganetespib for a variety of human cancers. In this regard, the sustained inhibition of the JAK2/STAT signaling axis achieved by ganetespib was more effective than that seen with the pan-JAK inhibitor P6, and ganetespib was uniformly more potent than the ansamycin based Hsp90 inhibitor 17-AAG in our assays.

**Figure 7:**
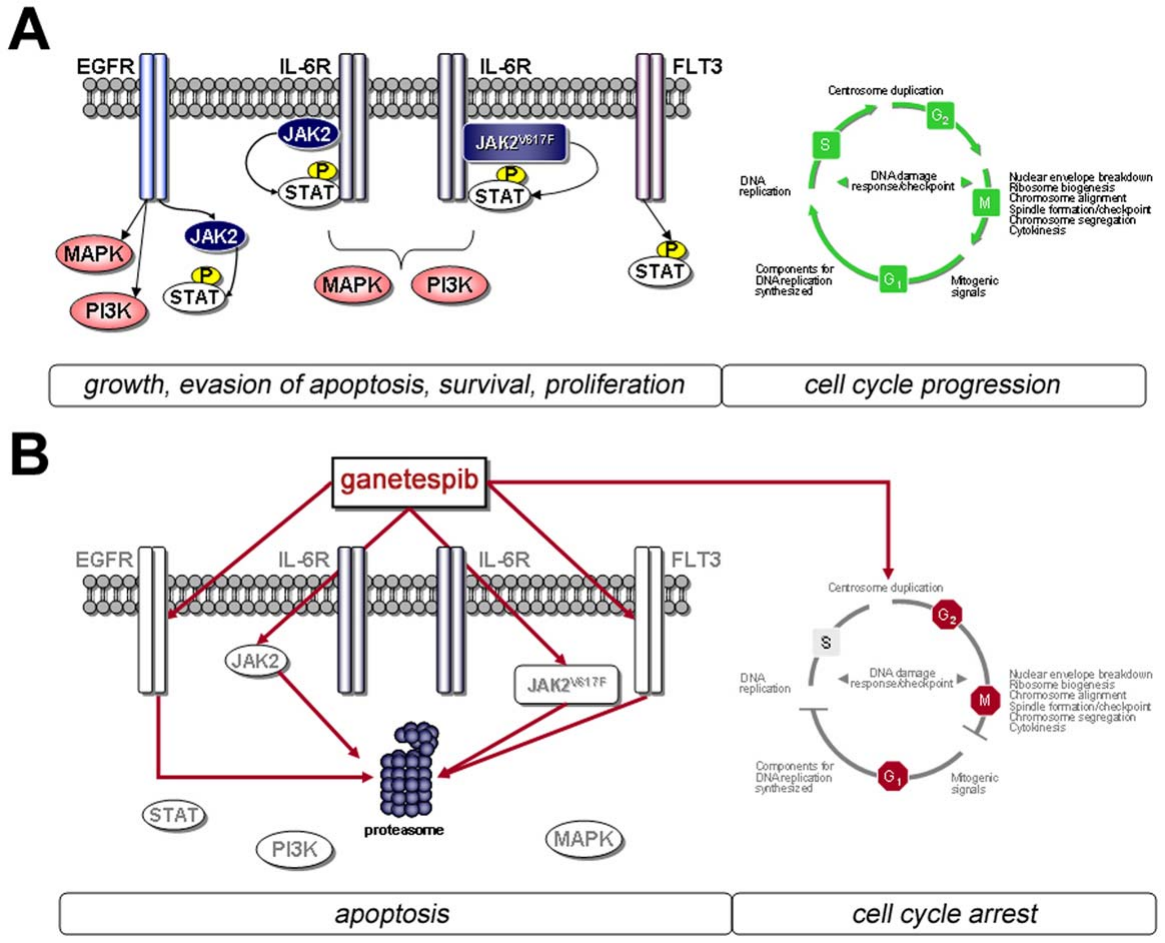
Multimodal activity of Hsp90 inhibition induced by ganetespib. Ganetespib exerts potent antitumor effects through perturbation of multiple signaling cascades, including the JAK/STAT signaling axis and cell cycle mediators. (A) The JAK/STAT pathway is a principal signaling mechanism for a wide array of cytokines and growth factors. Hyperactivation of this pathway, through ligand activated receptor tyrosine kinases (EGFR or FLT3), cytokine receptor mediated activation of JAK2, or activating mutations such as JAK2^V617F^, is often associated with oncogenesis. Regulation of the cell cycle involves a number of highly coordinated and essential processes, including checkpoint control and detection/repair of genetic damage, critical for correct progression and cell division. (B) Inactivation of Hsp90 by ganetespib results in the proteasome-mediated degradation of the upstream signaling components (indicated in grey) critical for STAT, MAPK and AKT activation, thereby resulting in growth inhibition. In addition, concomitant downregulation of key cell-cycle regulatory genes induced by ganetespib (as shown in Table 1) results in cell cycle arrest in the G_1_ and G_2_/M phases of the cell cycle, and subsequent loss of S phase.

While JAK2 mutation is a common means to stimulate oncogenic STAT activity, perturbations in other signaling networks, such as those mediated by EGFR, IL-6/IL-6R or FLT3, can also contribute to activated STAT signaling in cancer cells [2], [43]. Our results show that Hsp90 inhibition effectively disrupts these as well, with ganetespib potently degrading EGFR and blocking both IL-6- and FLT3-mediated activation of STAT proteins. Thus, while ganetespib directly imposes its pharmacological effects on Hsp90, the downstream consequences clearly involve a substantial array of client proteins and biochemical pathways. In this manner, Hsp90 inhibition by ganetespib can be viewed as a multi-nodal modality rather than a target-specific therapeutic approach, such as that engendered by a JAK2 or other kinase inhibitor (Figure 7).

In further support of this, while both ganetespib and P6 alter a common set of JAK/STAT targets, only ganetespib treatment exerted concomitant effects on the cell cycle regulatory machinery. In the leukemic cell data presented, exposure to ganetespib resulted in G_1_ and G_2_/M arrest, in part through the degradation of Cdk1 and atypical accumulation of cyclins A1 and B1. Further, S phase was abrogated. In addition to genes associated with DNA replication and the cell cycle, several components of the centrosome and spindle were affected at the transcriptional level by ganetespib, in agreement with previous findings that these components are synthesized in S phase and that Hsp90 is essential for centrosome assembly [44], [45]. Importantly, this was a general response in all cells studied, as we observed similar combinatorial effects on JAK/STAT inhibition with loss of cyclin-dependent kinase activity in AML, breast, gastrointestinal stromal, pancreatic and prostate tumor types.

Ganetespib also showed potent *in vivo* activity. In mice with established MV4-11 (STAT5-driven) xenografts, weekly administration of ganetespib significantly inhibited tumor growth in a dose-dependent manner. Moreover, a daily dosing schedule of ganetespib was also highly effective and resulted in significant tumor regression during drug administration. In this model, tumor growth reappears about a week after the drug treatment was stopped (for the high dose, 1×/week cohort). Importantly, our pharmacodynamic analysis showed that these tumor responses correlated with the degree and duration of STAT5 and Cdk1 protein loss induced by the varying dosing regimens. The tight linkage of STAT5 down-regulation with inhibition of tumor growth six hours after drug administration at either dose indicates the quick response of this signaling pathway to the drug administration. At the 150 mg/kg dose, STAT5 signaling, but not Cdk1 expression, returned by six days. The sustained loss of Cdk1 and other cell cycle proteins presumably maintains the cell cycle arrest and prevents growth from re-occurring between doses on the weekly schedule, even in the presence of the re-emergent STAT5 activity. Similarly, Cdk1 expression was suppressed longer in comparison to STAT5 at the 25 mg/kg dose, and is likely to account for the potent activity of ganetespib on the more frequent 5×/week regimen. These data strongly suggest that ganetespib administration on either schedule was sufficient to abolish both survival and cell growth signals long enough to prevent tumor growth. Because ganetespib presumably leads to the loss of even more client proteins, its potent antitumor activity likely reflects its combined impact on these additional target proteins as well.

In a system that more accurately mimics the pathology of leukemic disease, the efficacy of ganetespib was also evaluated in a disseminated disease model using HEL92.1.7 cells. Ganetespib effectively increased survival in this orthotopic model, more than doubling the median survival time of the mice. Prolonged survival was associated with dramatically reduced tumor burden in the bone marrow, as evidenced by significantly decreased infiltration of human leukemic cells and reduced spinal column metastases. Collectively, these data are consistent with a direct effect of ganetespib on leukemic cell growth *in vivo* and demonstrate the potential therapeutic utility of this compound for JAK2^V617F^-driven malignancies.

The causal relationship between constitutive JAK2 activity and neoplasia has resulted in the development of a variety of potent and selective JAK2 small molecule inhibitors [46]. However, emerging findings of the early phase trials are revealing added complexities in targeting this kinase in patients. Selective JAK2 inhibitors have been evaluated in patients with advanced MF and have shown considerable symptomatic benefit, including decreased splenomegaly and hematological improvement [23], [24]. However, these clinical responses were not consistently associated with a reduction in JAK2^V617F^ allelic burden [47], and patients showed benefit irrespective of the mutational status of JAK2. These findings suggest that clonal JAK2^V617F^-positive disease is not being fully targeted by these agents. A recent study [48] provides a possible mechanistic explanation for these observations. Their findings using a murine myeloproliferative neoplasm (MPN) model indicate that treatment with a selective JAK2 inhibitor attenuates the MPN phenotype by diminishing the myeloid progenitor population. However it had nominal effects on the JAK2^V617F^-positive, disease-initiating stem cells. If general, this has important implications for targeted JAK2 inhibitors as remitting rather than curative therapeutics due to the existence of a resistant reservoir of MPN-initiating cells [48]. Of particular relevance, the Hsp90 inhibitor PU-H71 was recently shown to reduce mutant allelic burden in murine MPN models [49], supporting the therapeutic rationale for Hsp90 inhibition in the treatment of JAK-driven disease due to its multifaceted impact in both cell populations.

In summary, ganetespib is a small molecule Hsp90 inhibitor with potent *in vitro* and *in vivo* activity in tumor cells harboring constitutively active JAK/STAT signaling. Due to its concomitant effects on this oncogenic signaling axis as well as cell cycle progression, ganetespib displays superior activity to both 17-AAG and the pan-JAK inhibitor P6 in terms of potency, duration of response, and pre-clinical efficacy. In light of these observations, further evaluation of the therapeutic utility of ganetespib for JAK/STAT-driven malignancies is warranted.

## Materials and Methods

### Cell culture

All cell lines were obtained from the ATCC (Rockville, MD, USA), with the exception of SET-2 cells which were purchased from the German Collection of Microorganisms and Cell Cultures (DSMZ, Germany). Cells were maintained and cultured according to standard techniques at 37°C in 5% (v/v) CO_2_ using culture medium recommended by the supplier.

### Reagents

Hsp90 inhibitors ganetespib and 17-AAG (both synthesized at Synta Pharmaceuticals, Inc.) and the JAK inhibitor Pyridone 6 (Calbiochem, Darmstadt, Germany) were dissolved in dimethyl sulfoxide (DMSO), aliquoted and stored at −20°C. All primary antibodies were purchased from Cell Signaling Technology (CST, Beverly, MA, USA) with the exception of JAK1 (Santa Cruz Biotechnology, Santa Cruz, CA, USA) and STAT5 (Epitomics, Burlingame, CA, USA).

### Cell viability assays

Twenty-four hours after plating, cells were dosed with the indicated compound or DMSO (0.3%) for 72 h. AlamarBlue (Invitrogen, Carlsbad, CA, USA) was added (10% v/v) to the cells, and the plates incubated for 3 h and subjected to fluorescence detection in a SpectraMax Plus 384 microplate reader (Molecular Devices, Sunnyvale, CA, USA). Data were normalized to percent of control.

### Western blotting

Cells were lysed with RIPA buffer (CST). Xenograft tumors (average volume of 100–200 mm^3^) were excised, cut in half, and flash frozen in liquid nitrogen. Each tumor fragment was lysed in 0.5 mL of lysis buffer using a FastPrep-24 homogenizer and Lysing Matrix A (MP Biomedicals, Solon, OH, USA). The lysates were clarified by centrifugation. Equal amounts of protein were resolved by SDS–PAGE and immunoblotted with indicated antibodies. The antigen-antibody complex was visualized and quantitated using an Odyssey system (LI-COR, Lincoln, NE, USA).

### Affymetrix gene expression analysis

Biotinylated aRNA was generated by *in vitro* transcription using the Affymetrix GeneChip Expression IVT labeling kit (Affymetrix, Santa Clara, CA, USA). Fifteen micrograms of labeled aRNA were fragmented and hybridized to Affymetrix GeneChip Human Genome U133 Plus 2 arrays and scanned using a GeneChip Scanner (Affymetrix). Array data were analyzed with the Affymetrix Expression Console Software utilizing the MAS5 algorithm. In order to generate a threshold for identifying probe sets that have large differences between the treated samples [1000 nM P6 (4 h) and 250 nM ganetespib (24 h)], and their controls, data from the four control arrays (DMSO only; two at 4 h, and two at 24 h) were used to create an expression level-dependent fold-difference envelope that reflects the increasing measurement variability as expression level decreases. This envelope, used in lieu of a fixed fold-difference criteria, was formed by identifying the x^th^ percentile expression-level difference (where x was large, typically 99.9%) at each mean expression level bin. To achieve this, data were used from the six possible comparisons from the four DMSO-only arrays. The resulting, smoothed threshold fold-difference envelope was then applied to the two compound-treated/control array pairs to identify those probe-sets that have large expression level differences between treatment and control. For hierarchical analysis, genes with greater than two fold changes in expression with 250 nM ganetespib were clustered using established algorithms in Cluster [50]. Annotation enrichment of the gene sets was performed using Database for Annotation, Visualization, and Integrated Discovery (DAVID) software [51].

### Flow cytometry

HEL92.1.7 cells were plated at 0.5×10^6^ cells/mL and treated as indicated. Cells were harvested and stained with propidium iodide using the BD Cycle TEST PLUS Reagent Kit (BD Biosciences, San Jose, CA, USA) according to the manufacturer's instructions. Twenty thousand cells were analyzed for their DNA content using a FACS Caliber cytometer (BD Biosciences).

### *In vivo* leukemia xenograft models

Eight-week-old female immunodeficient CB-17/Icr-*Prkdc^scid^*/Crl (SCID) mice (Charles River Laboratories, Wilmington, MA) were maintained in a pathogen-free environment, and all *in vivo* procedures were approved by the Synta Pharmaceuticals Corp. Institutional Animal Care and Use Committee (Protocol # AP-02.4-10). For the MV4-11 model, tumor cells were subcutaneously implanted in SCID mice as previously described [34]. Tumor volumes (V) were calculated by caliper measurements of the width (W), length (L), and thickness (T) of each tumor using the formula: V = 0.5236(LWT). Animals with 100–200 mm^3^ tumors were then randomized into treatment groups of 8 and i.v. dosed via the tail vein at 10 mL/kg body weight with either vehicle or ganetespib formulated in 10/18 DRD (10% DMSO, 18% Cremophor RH 40, 3.6% dextrose, 68.4% water). Tumor growth inhibition was monitored by tumor volume measurements twice weekly. As a measurement of *in vivo* efficacy, the %T/C value was determined from the change in average tumor volumes of each treated group relative to the vehicle-treated or itself in the case of tumor regression. Statistical significance was determined using a Kruskal-Wallis one-way ANOVA followed by the Tukey Test multiple comparison procedure.

For the HEL92.1.7 model, SCID mice were i.v. injected via the tail vein with 5×10^6^ cells in phosphate-buffered saline (PBS) on day 0. Implanted animals were then randomized into groups of 10 and i.v. dosed with either vehicle or ganetespib as above. Animals were weighed daily and removed from the study at the first sign of hind limb paralysis, which occurred in 100% of vehicle-treated animals. Median overall survival was estimated using the Kaplan-Meier method and the log-rank test (2-sided) for statistical significance. Spinal column tumor burden was visualized using hematoxylin and eosin stained tissue sections.

## Supporting Information

Figure S1 **Ganetespib differentially regulates genes associated with the cell cycle in addition to JAK/STAT signaling.** Genes with greater than two fold increases (red) or decreases (green) in expression with 250 nM ganetespib were clustered using established algorithms. TreeView was used to visualize these results and relevant subsets (Groupings A–D) are shown here. The genes associated with each Group are listed above the heat map. Group A contains genes rapidly and specifically induced by ganetespib (including heat shock-related genes). Group B contains genes repressed by P6 at both 4 and 24 hr (left column of gene names) or only 4 hr (right column). These genes are also inhibited by ganetespib at 24 hr; thus they can be considered JAK-response related. Group C identifies genes specifically repressed by ganetespib at 24 hr, most of which are cell cycle related. Group D includes those genes repressed both early and late by ganetespib but not P6.
(TIF)

Figure S2 **Ganetespib was well tolerated in the HEL92.1.7 disseminated leukemia model.** Cumulative average body weights showed minimal effects over the 3 week dosing period. Points represent the means and the error bars are the s.e.m.
(TIF)

Table S1 Inhibition of JAK2 activity by P6 or destabilization of JAK2 expression by ganetespib blocks STAT-target gene expression.
(DOC)
