# Supplementary figures and images for: Multifaceted Intervention by the Hsp90 Inhibitor Ganetespib (STA-9090) in Cancer Cells with Activated JAK/STAT Signaling
Source: PLoS One. 2011 Apr 14;6(4):e18552. doi: 10.1371/journal.pone.0018552 (PMC3077378; doi:10.1371/journal.pone.0018552)

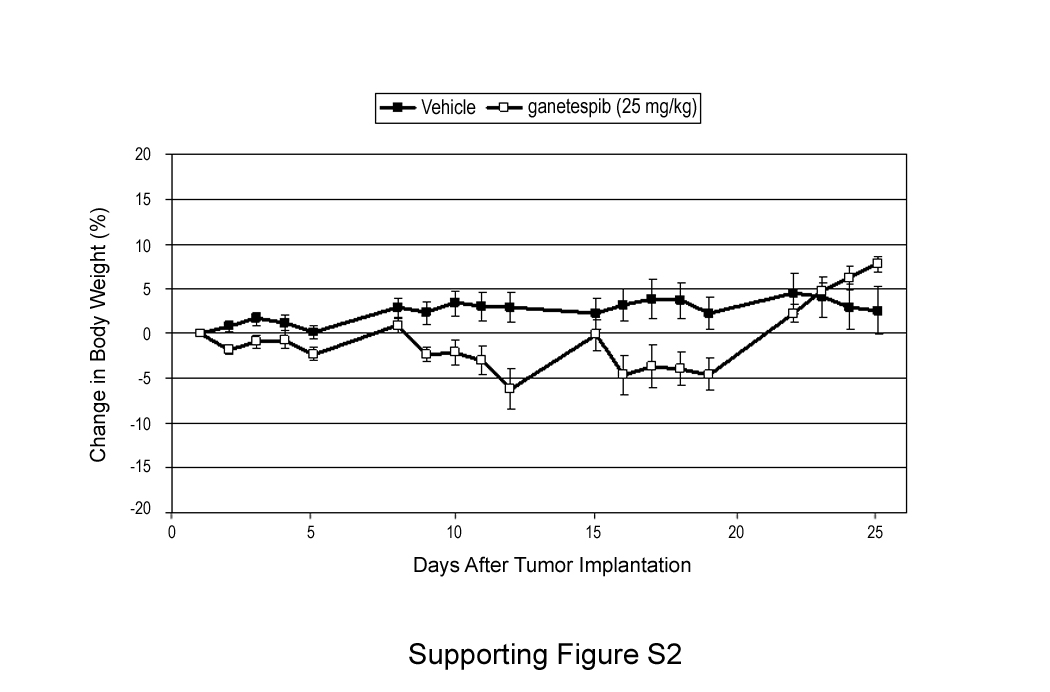

Supplement: Figure S2 — Ganetespib was well tolerated in the HEL92.1.7 disseminated leukemia model. Cumulative average body weights showed minimal effects over the 3 week dosing period. Points represent the means and the error bars are the s.e.m. (TIF) [file pone.0018552.s002.tif]
