# Supplementary material for: Multifaceted Intervention by the Hsp90 Inhibitor Ganetespib (STA-9090) in Cancer Cells with Activated JAK/STAT Signaling
Source: PLoS One. 2011 Apr 14;6(4):e18552. doi: 10.1371/journal.pone.0018552 (PMC3077378; doi:10.1371/journal.pone.0018552)
Supplement: Table S1 — Inhibition of JAK2 activity by P6 or destabilization of JAK2 expression by ganetespib blocks STAT-target gene expression. (DOC) [file pone.0018552.s003.doc]

**Supporting Table S1.** Inhibition of JAK2 activity by P6 or destabilization of JAK2 expression by ganetespib blocks STAT-target gene expression.

|  |  | Ganetespib | | P6 | |
| --- | --- | --- | --- | --- | --- |
|  |  | 4 hr | 24 hr | 4 hr | 24 hr |
| STAT Target Genes | PIM1 | 1 | -20 | -4 | -1 |
|  | PIM2 | -1 | -9 | -5 | -1 |
|  | SOCS3 | 1 | -5 | -9 | -2 |
|  | CDKN1A | 2 | -4 | -4 | -2 |
|  | SOCS1 | 1 | -2 | -2 | -2 |
|  | BCL3 | -1 | -2 | -4 | -1 |
|  | BCL2L1 | -1 | -1 | -2 | -1 |
|  | MCL1 | 1 | -1 | -1 | -1 |
| Stress Genes | HSP70A | 6 | 2 | -1 | 4 |
|  | BAG3 | 22 | 1 | -1 | -1 |
|  | HSP70B | 169 | 2 | -3 | -1 |

Values represent cycle threshold (Ct), normalized to HPRT. As expected, inhibition of Hsp90 by ganetespib results in upregulation of heat shock/stress proteins.
